# Effects of sinvastatin in prevention of vasospasm in nontraumatic subarachnoid hemorrhage: preliminary data

**DOI:** 10.1186/cc10922

**Published:** 2012-03-20

**Authors:** S Macedo, V Aguiar, PF Rosa, IT Ladeia, YK Castro, LA Ferreira, DR De Melo, LG Rezende

**Affiliations:** 1 São Jose do Avai Hospital, Itaperuna, Brazil

## Introduction

Some trials have shown that statins in the acute phase of aSAH reduce the incidence, morbidity and mortality of cerebral vasospasm. Independent of their cholesterol-lowering effect, statins have multiple biological properties, including downregulating inflammation and upregulating endothelial NO synthase. The purpose of this study is to evaluate the potential of sinvastatin (SVT) as prevention against vasospasm.

## Methods

We realized a prospective study, randomized, nonblind, with the use of 80 mg SVT (night) in the first 72 hours of the beginning of bleeding, and a control group that did not use SVT, for 21 days, between January and December 2008. Informed consent was obtained for all patients. CT scans were performed as control and another CT scan in patients with altered neurological signals. In the presence of changes suggestive of vasospasm or correlation in clinical and CT scans, the patients were taken for cerebral arteriography examination followed by an angioplasty procedure if necessary. Liver and renal function and LDL cholesterol were evaluated every 3 days. Exclusion criteria: liver and renal disease, pregnancy, elevation of serum transaminases (three times the value of normality), creatinine ≥2.5, rhabdomyolysis or CK total ≥1,000 U/l.

## Results

We excluded two patients with bleeding for more than 72 hours. There was no significant change in the levels of CK total, renal or liver function. We included 21 patients, 11 in the SVT group and nine in the control group. The mortality was eight patients (38%), six patients in the control group and two of the SVT group. Vasospasm was confirmed by cerebral arteriography examination in four patients in the control group and one patient in the SVT group. All patients that had a bad outcome (death) had Fisher IV scale.

## Conclusion

SVT at a dose of 80 mg was effective in reducing the mortality (18.1% against 66%) compared to the group that did not use SVT, and also decreased the incidence of cerebral vasospasm despite the APACHE II score being higher in the group that used SVT (14.3 vs. 10.7). There was less morbidity in the SVT group with an average Glasgow Outcome Scale of 3.25 vs. 2.1.
